# Assessment of operative times of multiple surgical specialties in a public university hospital

**DOI:** 10.1590/S1679-45082017GS3902

**Published:** 2017

**Authors:** Altair da Silva Costa

**Affiliations:** 1 Hospital Israelita Albert Einstein, São Paulo, SP, Brazil.; 2 Escola Paulista de Medicina, Universidade Federal de São Paulo, São Paulo, SP, Brazil;; Hospital São Paulo, Universidade Federal de São Paulo, São Paulo, SP, Brazil.

**Keywords:** Quality indicators, health care, Operating rooms, Operative time, Health Information Management, Time management, Specialties, surgical

## Abstract

**Objective:**

To evaluate the indicators duration of anesthesia, operative time and time patients stay in the operating rooms of different surgical specialties at a public university hospital.

**Methods:**

It was done by a descriptive cross-sectional study based on the operating room database. The following stages were measured: duration of anesthesia, procedure time and patient length of stay in the room of the various specialties. We included surgeries carried out in sequence in the same room, between 7:00 a.m. and 5 p.m., either elective or emergency. We calculated the 80th percentile of the stages, where 80% of procedures were below this value.

**Results:**

The study measured 8,337 operations of 12 surgical specialties performed within one year. The overall mean duration of anesthesia of all specialties was 178.12±110.46 minutes, and the 80th percentile was 252 minutes. The mean operative time was 130.45±97.23 minutes, and the 80th percentile was 195 minutes. The mean total time of the patient in the operating room was 197.30±113.71 minutes, and the 80th percentile was 285 minutes. Thus, the variation of the overall mean compared to the 80th percentile was 41% for anesthesia, 49% for surgeries and 44% for operating room time. In average, anesthesia took up 88% of the operating room period, and surgery, 61%.

**Conclusion:**

This study identified patterns in the duration of surgery stages. The mean values of the specialties can assist with operating room planning and reduce delays.

## RESUMO

**Objetivo:**

Avaliar os indicadores de tempo da anestesia, da operação e da permanência do paciente em sala de diversas especialidades do centro cirúrgico de um hospital universitário.

**Métodos:**

Foi realizado em estudo descritivo transversal a partir da base de dados do centro cirúrgico e mensuradas as seguintes etapas: duração de anestesia, tempo do procedimento e tempo de permanência do paciente em sala das diversas especialidades. Foram incluídas as operações realizadas em sequência na mesma sala, das 7h às 17h, eletivas ou de urgências. Realizamos o calculo do percentil 80 da duração das etapas, onde 80% dos procedimentos ficaram abaixo deste valor obtido.

**Resultados:**

O estudo incluiu 8.337 operações realizadas no período de 1 ano de 12 especialidades cirúrgicas. A média geral da duração da anestesia de todas as especialidades foi de 178,12±110,46 minutos, e o percentil 80 foi de 252 minutos. A média do tempo operatório foi 130,45±97,23 minutos, e o percentil 80 foi de 195 minutos. A média do tempo total do paciente em sala operatória foi de 197,30±113,71 minutos, e o percentil 80 foi de 285 minutos. A variação da média geral em relação ao percentil 80 foi de 41% na anestesia, 49% nas operações e 44% no tempo de sala. Na média geral, a anestesia ocupou 88% do tempo de sala e a operação, 61%.

**Conclusão:**

Este estudo identificou padrões nas durações das etapas das operações. A informação das médias históricas das especialidades pode auxiliar no planejamento do centro cirúrgico e diminuir os atrasos.

## INTRODUCTION

The World Health Organization estimated that 312 million surgical procedures were performed in the world in 2012.^1^ In the United States, there were approximately 8 million hospital admissions for procedures in the operating room in 2012, amounting to hospital cost of approximately US$ 168 billion.^2^ The operating room is one of the most complex structures of the hospital system, and generally more than 60% of admitted patients need some surgical intervention.^3-5^ For the operating room management, the basis is the surgical suite, and evaluation can be divided into duration of anesthesia, operative time, operating room time, and operating room preparation time. Anesthesia duration is divided into four moments: anesthetic induction, maintenance, awakening and recovery. The operative time consists of dieresis, hemostasis, exeresis, and suture. Operating room time includes patient stay, from arrival to exit. Operating room preparation time (turnover) is the time between one patient leaving and another entering, including cleaning and replacing the necessary material.^6^ The lengthiest stage during a procedure is anesthesia. Quite often, the planning of the use of operating rooms do not happen in the expected way, but with delays, since the rooms must be shared by multiple specialties.^6,7^

The decision to treat a patient through an intervention is based on knowledge acquired through scientific evidence. Paradoxically, the prediction of procedure duration is estimated through the surgeon’s experience:^6-8^ all operating room scheduling grids depend on reliable estimations from the several teams that schedule the procedures stochastically (from Greek *stokhastikós,* “able to estimate, predict”).^8,9^ When an operation takes longer than predicted, all subsequent procedures become progressively delayed.^7^

The surgical schedule can be done in two ways:^7,8,10,11^ with a stochastic estimation of procedure duration, or based on measurable data from the team, such as the duration of the specific procedure, historical performance (delays), available technology, local structure, and the ability to solve adverse events. Surgeons generally take such data into account and empirically ground their decision to estimate the duration of a procedure. The ability to estimate the duration of a procedure depends on professional wisdom (knowledge and experience) and delays are frequent in most public and private operating rooms.^7,10,11^ Unfortunately, we found few national data in the searched literature about procedure and anesthesia duration of several specialties.

## OBJECTIVE

To evaluate indicators of operative times of several specialties in the operating room of a public university hospital.

## METHODS

This was a descriptive cross-sectional study carried out from databases of the information technology system of the operating room of a teaching hospital from a federal university. The sample was composed by operations performed between January 2011 and January 2012. We included surgical interventions that took place during routine hours of the operating room, between 7:00 a.m. and 5 p.m., elective or emergency procedures. Exclusion criterias were: procedures with incomplete data in the system; those that started after 5:00 p.m. or were performed on weekends or holidays. The university hospital’s operating room had 19 active operating rooms.

The process that took place in the operating rooms was divided into seven stages to collect the respective variables: (1) patient stay in the operating room or operating room time; (2) time between the patient’s entrance in the room and beginning of anesthesia (anesthetic induction); (3) time between anesthetic induction and beginning of operation; (4) duration of anesthesia; (5) duration of procedure; (6) time between the end of the procedure and the end of anesthesia (awakening); (7) time between ending of anesthesia and patient exit from the room. The collected data were submitted to a descriptive analysis.

We calculated the 80th percentile (P80) of the measured times and their variation in relation to the mean. With this information, we verified the value of 80% of the duration of the following stages: anaesthesia, operation and operating room time. This data was more pragmatic than the time average of the stages. To calculate the variation between mean and P80, we used the following formula: (P80 value/mean value) -1. We also measured the proportion of the stages anesthesia and operation in relation to operating room time – these values were expressed in percentages.

The project was approved by the Research Ethics Committee of the organization under protocol 165.292/2012, CAAE: 07233312.9.0000.5505, with authorization from the hospital’s operating room coordination. Informed consent was waived due to the applied research method.

## RESULTS

A total of 12,114 procedures were performed between January 2011 and January 2012. Of those, the study included 8,337 procedures (68.82%) and 3,777 (31.18%) were excluded for incomplete data.

**Figure 1:**
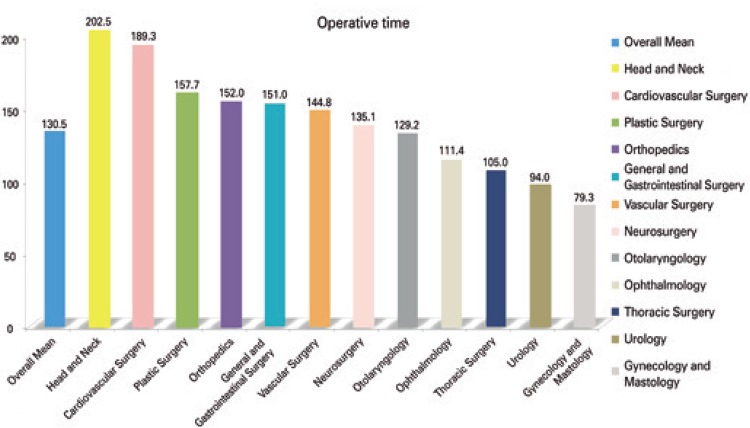
Mean operative time per specialty

The operative times varied between 1.2 minutes and 14.6 hours. The complexity of the procedures also showed great variation, and included some simples ones, as catheter removal, nevus resection, and other more complex as transplants. Orthopedics was the specialty with the highest number of procedures – 16.6% of total. Table 1 shows the specialties with the respective number of analysed procedures, as well as duration of anesthesia, operation and operating room time (Figure 1).

**Table 1:** Number of procedures analyzed by specialty, with the respective percentage, mean and standard deviation (in minutes)

Specialties	Number of the surgeries n (%)	Duration of the anesthesia	Duration of the surgery	Operating room time
Orthopedics	1,385 (16.6)	207.52±104.96	151.95±92.45	228.18±110.30
General and Gastrointestinal Surgery	1,324 (15.9)	200.01±110.38	150.95±98.27	218.01±113.73
Gynecology/ Mastology	1,116 (13.4)	109.95± 89.34	79.32±79.43	128.46±92.46
Urology	959 (11.5)	135.44± 86.85	94.00±77.30	153.99±90.24
Plastic Surgery	779 (9.3)	205.73±106.92	157.72±97.44	225.75±111.74
Otolaryngology	719 (8.6)	176.54±96.48	129.23±86.64	194.88±98.54
Ophthalmology	669 (8.0)	153.54±94.85	111.38±86.19	172.02±97.26
Neurosurgery	447 (5.4)	199.27±104.05	135.06±92.12	218.45±105.66
Vascular Surgery	314 (3.8)	193.53±114.88	144.78±102.34	215.36±117.57
Head and Neck	280 (3.4)	257.63±145.36	202.45±133.31	279.19±147.67
Thoracic Surgery	231 (2.8)	163.39±106.15	104.95±82.58	183.64±107.09
Cardiovascular Surgery	114 (1.4)	268.00±141.89	189.34±123.76	283.25±140.52
Overall mean	8,337 (100)	178.12±110.46	130.45±97.23	197.30±113.71

**Figure 2:**
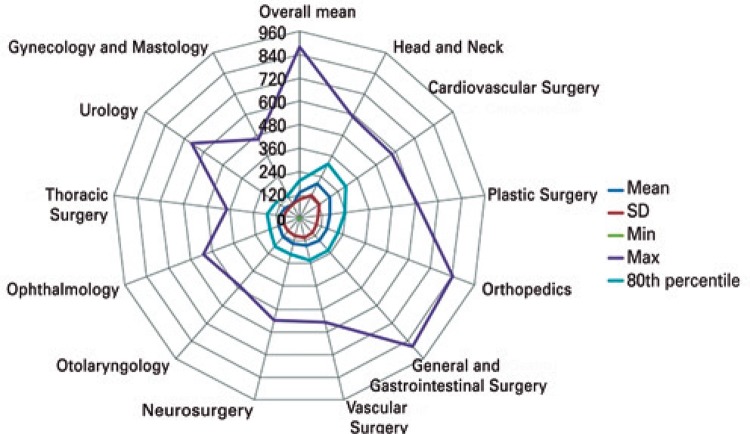
Duration of the operative stage in general and per specialty

SD: standard deviation; min: minimum; max: maximum.

**Figure 3:**
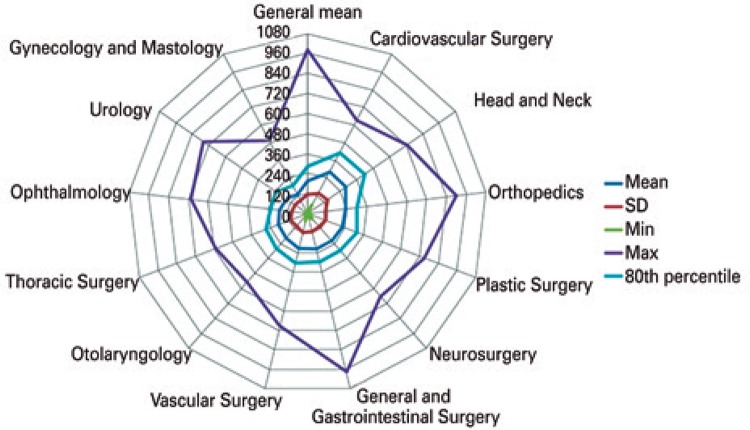
Operating room stay time in general and per specialty

SD: standard deviation; min: minimum; max: maximum.

With the parameters observed in the descriptive analysis, we verified the variation between P80 and the mean duration of procedures in the different specialties (Table 2). In the overall mean, 80% of anesthesia times had duration of up to 41% above the mean (252 minutes). In the operations, the variation between the mean and P80 was up to 49% (195 minutes; figure 2) and, in the operating room time, it was as high as 44% (285 minutes; figure 3).

**Table 2:** Mean, 80th percentile and variation between them, in the stages anesthesia, operative time and operating room time (in minutes)

Specialties	Duration of anesthesia			Operative time			Operating room time		
		
	Mean	P80	Variation (%)	Mean	P80	Variation (%)	Mean	P80	Variation (%)
Orthopedics	207.5	282.0	36	152.0	210.0	38	228.2	300.0	31
General and Gastrointestinal Surgery	200.0	282.0	41	151.0	222.0	47	218.0	300.0	38
Gynecology and Mastology	110.0	180.0	64	79.3	132.0	66	128.5	192.0	49
Urology	135.4	192.0	42	94.0	144.0	53	154.0	225.0	46
Plastic Surgery	205.7	294.0	43	157.7	237.0	50	225.7	316.2	40
Otolaryngology	176.5	255.8	45	129.2	193.2	50	194.9	280.2	44
Ophthalmology	153.5	195.0	27	111.4	162.0	45	172.0	225.0	31
Neurosurgery	199.3	279.8	40	135.1	189.0	40	218.5	294.0	35
Vascular Surgery	193.5	278.4	44	144.8	222.0	53	215.4	307.2	43
Head and Neck	257.6	379.8	47	202.5	312.6	54	279.2	417.0	49
Thoracic Surgery	163.4	240.0	47	105.0	168.0	60	183.6	264.0	44
Cardiovascular Surgery	268.0	409.2	53	189.3	291.0	54	283.3	414.0	46
Overall mean	178.1	252.0	41	130.5	195.0	49	197.3	285.0	44

P80: 80^th^ percentile.

Regarding the variation between the mean and the maximum value of the respective times, the difference was much bigger, but only 20% of procedures fit this scenario. An emergency exploratory laparotomy was the longest procedure among all analysed, with anesthesia duration of 980 minutes, that is, 4.2 times the mean, and the operation took 880 minutes – 5.7-fold the mean. Among the specialties, the biggest mean variation in relation to the maximum value was found in urology, in the surgery stage – 7.18 times, or 718% (Figure 2). This can be observed in figures 2 and 3 - the highest values of all stages were found in general surgery and gastrointestinal surgery. Osteosynthesis was the most frequently procedure performed (726 operations), with a mean operative time of 174.9±92.6 minutes, P80 of 228 minutes, and variation between the mean and P80 of 30%. The mean duration of anesthesia was 234.2±102.8 minutes, P80 was 297 minutes, and variation was 27%. The mean operating room time was 255±107.2 minutes, P80 was 312 minutes, and variation was 22%.

All seven stages were measured in the descriptive analysis of the 8,337 procedures (Table 3). At the end of the operation, the sum of the time for the patient to awaken plus operating room exit time was 31.4 minutes, with a P80 of 61 minutes. The sum of the mean operating room entrance time, plus anesthetic induction was 48.4 minutes, with a P80 of 69 minutes.

**Table 3:** Duration times of operating room stages

	Operating room stages	Mean	P80
1	Operating room time	197.3	285
2	Interval between patient arrival and beginning of anesthesia	12.3	15
3	Time between the beginning of anesthesia and beginning of surgery	36.1	54
4	Duration of anesthesia	178.1	252
5	Operative time	130.5	195
6	Interval between the end of the surgery and the end of anesthesia	11.6	28
7	Interval between the end of anesthesia and patient exit from the OR	19.8	33
	Awakening time + time to exit the OR (6+7)	31.4	61

OR: operating room.

**Figure 4:**
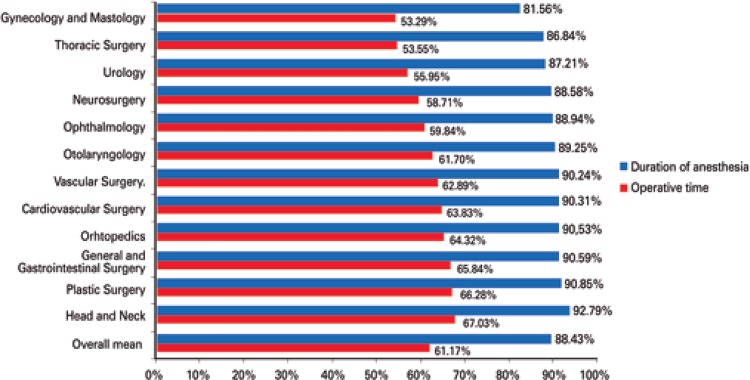
Duration of anesthesia and operative time in relation to the patient’s operating room stay time in general and per specialty

About the patient’s stay in the operating room, the anaesthesia mean time took up 88.4% of the operating room time, and the operation took 61.1%. After the procedure was finished, the sum of the awakening time and patient exit from the operating room amounted to 19.8% of the operating room time. The sum of the interval between the patient’s arrival in the operating room and beginning of anesthesia, plus the interval between the end of the anesthesia and patient exit from the operating room amounted to, in average, 11.6% of operating room time. Anesthetic induction and awakening corresponded to 27.3% of operating room time. In some specialities, such as thoracic surgery, gynecology and mastology, neurosurgery and ophthalmology, the procedure time took less than 60% of operating room time (Figure 4).

## DISCUSSION

In developed countries the annual healthcare expenditure per capita was over US$ 1,000.00; the operation rate was 11,110 procedures per 100,000 individuals. In Hungary, *e.g*., 23,369/100,000 procedures were performed in 2004. In developing countries, where the annual expenditure is below US$ 100.00 per capita; the operation rate was 295/100,000. In Ethiopia, the rate was 148/100,000.^1,12^ The mean annual operating rate in the world was 4,016/100,000 individuals. Currently, it is estimated that more than 230 million operations are performed per year in the world.^1,2,12^

Considering the significant number of operations performed worldwide, gathering appropriate information is essential to understand operating room characteristics, identify possible issues, and make it more efficient.^3^ The collection of data in our sector was performed by the nursing staff through a hand filled form. About 30% of the obtained information was incomplete, especially due to the absence of data in certain fields of the form, or illegible data. The collection system should be optimized and digitalized.^3,5,13,14^

In this study, we evaluated operations performed by 12 surgical specialties in the period of one year, amounting to 8,337 procedures. Each specialty has its characteristics and singularities – but even with such variability, operating room management can be efficient, dynamic and avoid waste.^11,13^ Information on operating room management and on the number of operations performed in Brazil are limited.^3^ We analysed over 8,000 procedures with complete information, which allowed us to obtain the mean duration of specific procedures. Despite unpredictable events and complications that may occur during an operation, management and planning on operating room time must be done based on that information.^5,13,14^ The duration of a procedure depends on patient individuality, particular characteristics of the disease, and the surgeon’s skill - the mean duration of the procedure does contemplate such variations.^3^ Mean operative time was similar to the reference standard performance found in the literature – approximately 120 minutes.^11^

As expected, the specialties present differences between themselves and the general mean, and these differences were evident in our results. The calculation of P80 made it possible to verify that most operations presented their own singularities, even within the same specialty. For example, in orthopedics (specialty with the greatest number of procedures), the mean operative time was 23 minutes lower than the mean of the most frequently performed procedure, osteosynthesis. Moreover, the P80 of general orthopedics and osteosyntheses operative times presented an 18 minutes difference. With this level of information, scheduling a procedure could be more precise, due to the information on the surgery and type of operation. For example, if the orthopedic surgeon schedules an osteosynthesis with an estimated duration of approximately 120 minutes, the system already detects that such procedure has a historical mean of 174 minutes. Moreover, considering that it may varies 30% and in 80% of the cases, it could take up to 228 minutes. Procedure scheduling based on the surgeon’s estimation method, paired with historical time factors, provides a statistical model to adjust these estimates with more precision. For example, in other study with 8,093 procedures, the authors improved estimations by 39 minutes per procedure.^15^ Other authors have analysed 116,599 operations from several specialties, with a overestimation of procedure scheduling of up to 30 minutes.^6^

The availability of historical means of the duration of operation stages has provided surgeons with the possibility of comparing their historical operative times for a certain procedure. Similar techniques were suggested in other articles, which examined different metrics and references (benchmarks), considered important for performance and general use of the operating room.^5-9,13-15^ A specialty historical duration mean may be useful as internal control. If the surgeon believes his next patient is similar to other recent ones, he may use his own history as a basis for estimation; or, if he believes his next patient is more complex, he may compare it to the historical duration mean and then estimate duration.^6^ The estimates can be potentially optimized within specialties to allow better allocation of resources in the operating room, such as time.

The interaction between the surgical team and the hospital is crucial and dynamic. The complexity and singularity of each patient must be considered when scheduling the procedure and estimating its duration.^6,7,10,16^ Surgery scheduling and the operating room schedule map must follow the rules established by the hospital board. These efforts, resources and advanced software are of no use if some surgeons do not comply with the rules or have privileges that allow them to make last minute changes in elective procedures.^14-16^ Elective and emergency surgeries also interfere in the estimation of procedure duration, and those of emergency character tend to take longer than predicted.^16^ It is notorious that the operating room must contemplate possible emergency procedures, but it is essential that elective surgery changes be an exception and avoided as much as possible.^5-11^ Alterations have a cascading impact on other previously booked procedures and, generally, these alterations include an increase in operating room time or in complexity.^14-16^ The type of complexity is important for the organization of operating suites, since the estimation for lengthy operations is less precise than short ones. Simpler procedures with shorter durations, of approximately 60 minutes, have smaller variations and risks and are more predictable.^7,10^ The duration of complex procedures, such as brain surgery, transplants, hepatectomies, tends to be overestimated.^6^ To estimate the duration of a procedure, one must also take into account the patient’s singularities, such as body mass index, previous procedures, associated diseases, neoplasm, systemic infection, localized or advanced diseases, physical status classification as I to IV, according to the American Society of Anesthesiologists, mechanical ventilation, and type of anesthesia.^7,10,11^

The challenges arise, for example, when the procedure’s actual time is underestimated. Delays accumulate in a cascading effect with subsequent operations, generating waste of time and resources, staff fatigue, and dissatisfied health professionals and patients. Therefore, surgery time estimation has a series of cascading effects, not only on the procedure itself, but also on areas such as the post-anesthetic recovery unit, intensive care, and inpatient units. On the other hand, if the operative time is overestimated, the operating room might become idle and underused, which also results in waste of resources.^6^ Operations involve a number of other professionals, specialties, and resources, such as pharmacy, central sterilization and supplies department, clinical engineering, and anesthesia, all of which depend on this schedule to work properly.

Regarding the patient’s length of stay in the operating room, anesthesia took up, in average, the longest operating room time (88.4%), and the operation itself took up 61.1%. There is a tendency to underestimate the anesthesia time for the total process time. In general, we can base anesthesia time on the operative time, with an addition of 33% (instead of a fixed number of minutes) and, thus, better predict the total operating room time.^6,14,16^

Moreover, some actions may be taken to start operating room, preparation for the subsequent surgery right at the end of the current one, before the patient awakens. Our study has shown that the mean awakening time, added to exit time, was 31.4 minutes - 19.8% of operating room time. Thus, it is possible to use 20% of operating room time to start preparations for the next procedure.^9^ Processes must be parallel, not sequential. The knowledge of operating room stages and their measurements are essential to the planning of the sector. Basing the entire operating room organization only on estimates by the surgical teams can results in more frequent unforeseen events.^14^

In general, operative time estimation by the surgeon is a strong predictor of the total operating room time, but it is still a subjective evaluation. A potential problem is the reproducibility of this estimate, considering it is an opinion and not an objective factor.^7,14-16^ Significant improvements in operating room use are possible. Further studies should be carried out to identify causes and find new solutions, considering the operating room is present in most – if not all – Brazilian hospitals.

## CONCLUSION

This study identified duration patterns in surgeries by several specialties and their variations in the stages: operative time, anesthetic time, and operating room stay. The indicators offer a tool and are an opportunity to improve the efficiency of operating room time management and scheduling. The hospitals are able to provide the historical mean of procedure stages to improve surgeons’ estimations.
